# Novel *SMAD3* Mutation in a Patient with Hypoplastic Left Heart Syndrome with Significant Aortic Aneurysm

**DOI:** 10.1155/2014/591516

**Published:** 2014-03-03

**Authors:** Kristi K. Fitzgerald, Abdul Majeed Bhat, Katrina Conard, James Hyland, Christian Pizarro

**Affiliations:** ^1^Nemours Cardiac Center, Nemours/Alfred I. duPont Hospital for Children, 1600 Rockland Road, Wilmington, DE 19803, USA; ^2^A Department of Pathology, Nemours/Alfred I. duPont Hospital for Children, Wilmington, DE 19803, USA; ^3^Anatomy and Cell Biology, Thomas Jefferson University Hospital, Philadelphia, PA 19107, USA; ^4^Connective Tissue Gene Tests, Allentown, PA 18106, USA

## Abstract

Aneurysms-osteoarthritis syndrome (AOS) caused by haploinsufficiency of *SMAD3* is a recently described cause of syndromic familial thoracic aortic aneurysm and dissection (TAAD). We identified a novel *SMAD3* mutation in a patient with hypoplastic left heart syndrome (HLHS) who developed progressive aortic aneurysm requiring surgical replacement of the neoaortic root, ascending aorta, and proximal aortic arch. Family screening for the mutation revealed that his father, who has vascular and skeletal features of AOS, and his brother, who is asymptomatic, also have the pathogenic mutation. This is the first case report of a *SMAD3* mutation in a patient with hypoplastic left heart syndrome. This case highlights the importance of genetic testing for known causes of aneurysm in patients with congenital heart disease who develop aneurysmal disease as it may significantly impact the management of those patients and their family members.

## 1. Introduction

Familial thoracic aortic aneurysm can be divided into syndromic and nonsyndromic forms. While abdominal aortic aneurysm generally occurs sporadically, thoracic aortic aneurysm and dissection (TAAD) is inherited in an autosomal dominant manner with decreased penetrance and variable expression [1]. The genes causing syndromic and nonsyndromic forms of TAAD encode proteins that compose the structural components associated with connective tissue, key members of the TGF-*β* signaling pathway, or components of the contractile unit of smooth muscle cells. The genetic etiology of nonsyndromic causes of familial TAAD is largely unknown; however, several genes including, *MYH11*, *ACTA2*, and *MYLK* have been implicated [2–4]. The genetic cause of syndromic forms of TAAD include *FBN1*, the cause of Marfan syndrome, *SLC2A10*, the cause of arterial tortuosity syndrome, and *TGF*β*R1*, *TGF*β*R2*, and the recent identification of *TGF*β*2*, all of which cause Loeys-Dietz syndrome [5–9].

In 2011 *SMAD3*, was shown to cause a new syndromic form of thoracic aortic aneurysm and dissection. The features of this condition included early onset osteoarthritis in the majority of patients and the authors proposed the name aneurysms-osteoarthritis syndrome (AOS) [10]. In addition to aneurysm and dissection, early osteoarthritis, and other systemic findings, congenital heart disease including persistent ductus arteriosus, atrial septal defect, pulmonary valve stenosis, atrial fibrillation, and bicuspid aortic valve have also been observed in patients with defects in *SMAD3* [11]. *SMAD3* encodes an intracellular member of the TGF-*β* signaling pathway that activates or represses gene transcription. Heterozygous mutations in *SMAD3* lead to increased expression of several components of the TGF-*β* pathway including phosphorylated SMAD2, total *SMAD3*, *TGF*β*1*, and connective tissue growth factor [10].

We present a 14-year-old boy born with hypoplastic left heart syndrome (HLHS) who developed significant aneurysm of the neoaorta and proximal arch after completed, staged palliation and who was found to have a novel, pathogenic *SMAD3* mutation. Further testing in the family revealed additional at risk family members and who were offered appropriate cardiovascular and orthopedic screening.

## 2. Case Presentation

**Figure 1:**
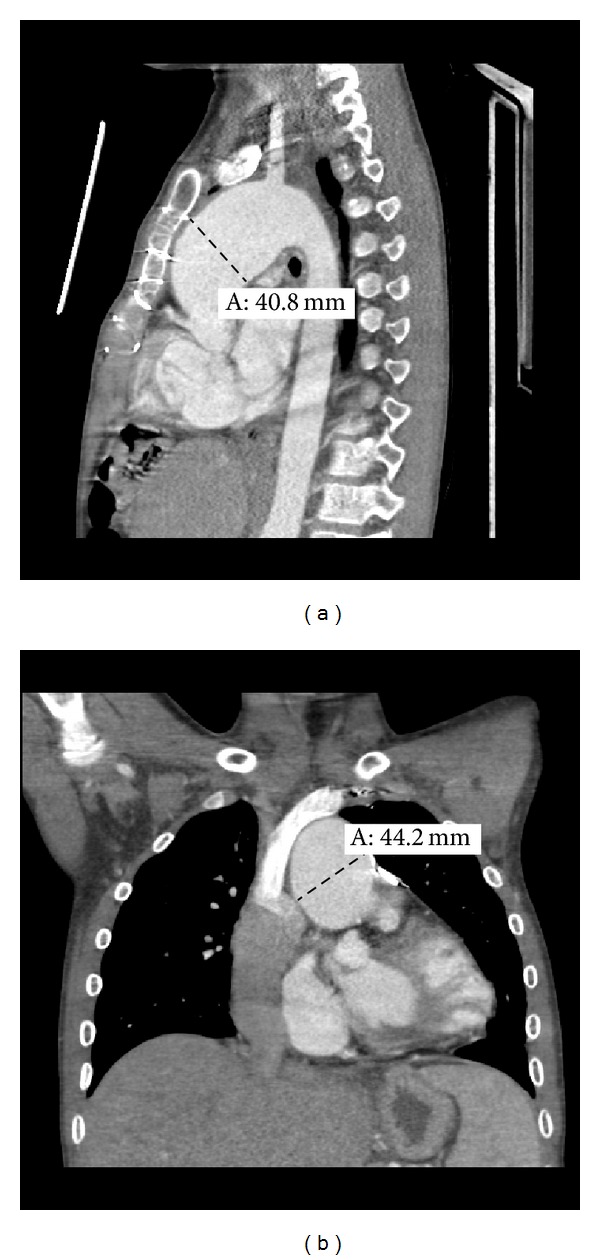
Optiray contrast-enhanced computed tomography images of the chest with sagittal (a) and coronal (b) reconstructions in the 14-year-old proband revealing aneurysmal dilation of the ascending aorta.

**Figure 2:**
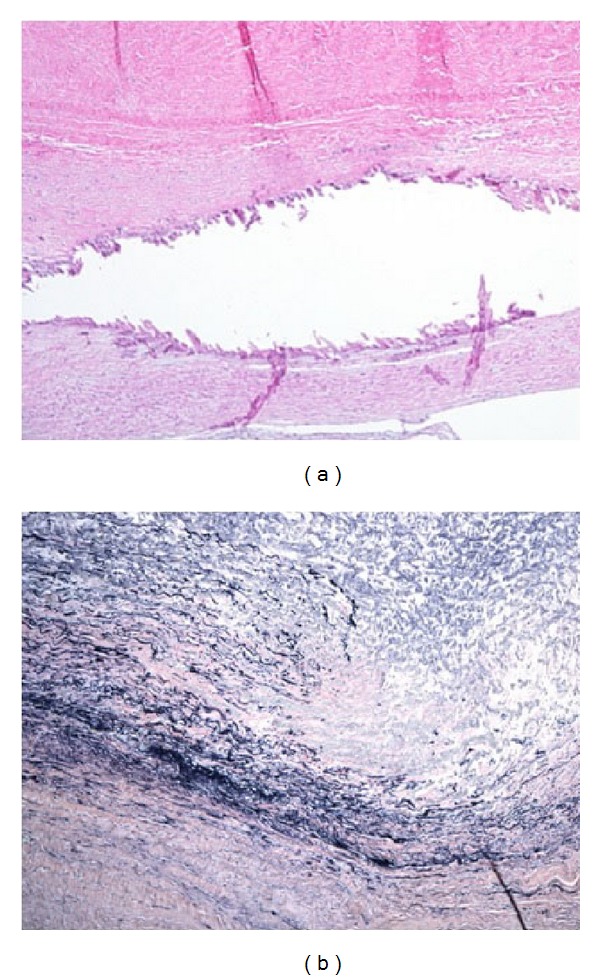
(a) Ascending aorta: medial fibrosis and dystrophic calcification (Hematoxylin and Eosin) and (b) fragmentation and disorganization of the elastic fibers (Verhoeff Van Gieson's elastic stain).

The proband, a 14-year-old male, was conceived via in vitro fertilization secondary to infertility and was diagnosed prenatally with hypoplastic left heart syndrome with mitral valve hypoplasia and aortic valve stenosis. He underwent palliative staged reconstruction including modified Fontan procedure. He developed significant aneurysmal dilatation of his reconstructed ascending aorta and neoaortic root and regurgitation of his native pulmonic valve. He underwent partial replacement of his neoaortic root with a 24 mm Hemashield graft at an outside institution. Due to significant residual neoaortic regurgitation, he underwent neoaortic valve replacement with a 25 mm On-X prosthetic. Subsequently, over a few years, he exhibited progressive dilatation of the remaining neoaorta reaching a maximal dimension of 5.2 cm. He presented to the emergency room with history of severe chest pain radiating to the neck following exercise. Computed tomography scan ruled out the presence of an aneurysmal rupture or dissection but revealed a fusiform aneurysmal dilatation of the ascending aorta with greatest dimensions of the ascending aorta of 4.1 cm (AP) × 4.4 cm (RL) (Figures 1(a) and 1(b)). He underwent aortic root, ascending aorta, and partial transverse aortic arch replacement with a 26 mm woven Dacron (Hemashield) graft, reimplantation of the mechanical prosthesis into the graft as well as reimplantation of the proximal native ascending aorta on the side of the graft. Microscopic examination of the resected ascending aorta and native pulmonary artery showed elastic artery with mucopolysaccharide rich areas and fibrosis with fragmentation and disorganization of the elastic fibers. Additionally, the aorta showed dystrophic calcification (Figures 2(a) and 2(b)).

**Figure 3:**
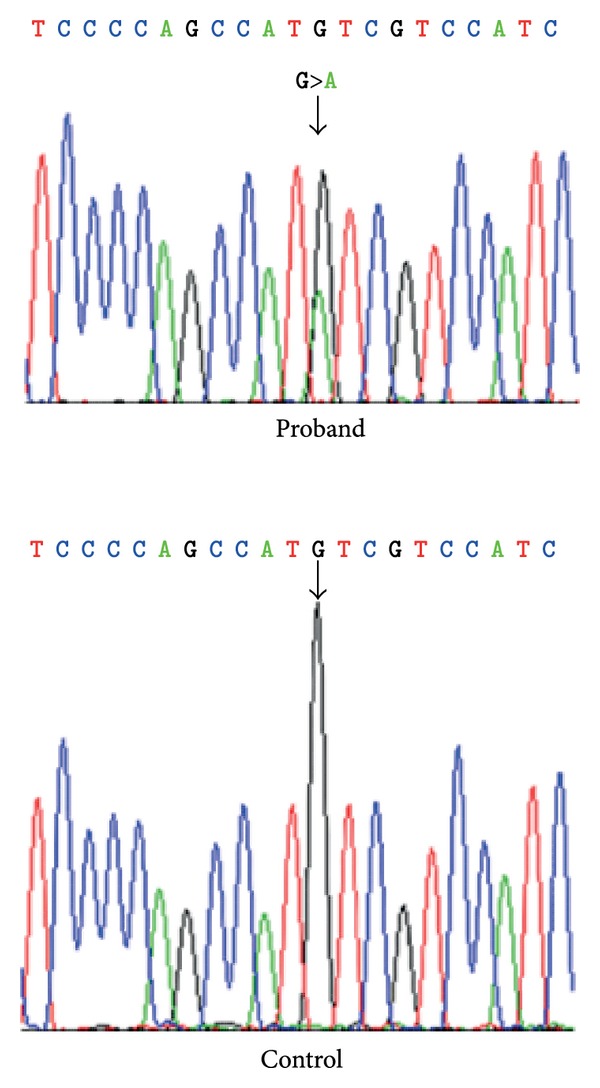
Sanger sequencing chromatogram revealing the pathogenic *SMAD3* mutation, c.3G>A (p. Met1Ile) in the proband versus the normal sequence in a control.

The proband underwent sequencing of genes known to cause thoracic aortic aneurysm and dissection. Next generation sequencing followed by Sanger sequencing revealed that the proband has a novel, pathogenic *SMAD3* mutation, c.3G>A (p. Met1Ile) (Figure 3). First degree relatives were screened for the *SMAD3* mutation. The proband's 46-year-old father and 9-year-old brother were also found to have the *SMAD3* c.3G>A mutation. The affected family members underwent transthoracic echocardiography and magnetic resonance angiography (MRA) of the brain, neck, chest, abdomen, and pelvis; the proband's MRA was normal. The father, now 46 years, has intervertebral disc degeneration of his cervical spine and osteoarthritis of his knee. Echocardiogram revealed aortic root dilation with a diameter of 4.0 cm and mitral valve prolapse with mild to moderate mitral regurgitation. MRAs of the neck, chest, abdomen, and pelvis were normal. The proband's brother had a normal echocardiogram and total body MRA. His past medical history is significant for left sided inguinal hernia requiring surgical repair. Of note, the proband has no evidence of osteoarthritis. Additional screening for the *SMAD3* mutation in extended relatives was recommended.

## 3. Discussion

In this report, we describe a proband with HLHS who developed significant aortic aneurysm in whom clinical sequencing for genetic causes of TAAD revealed a pathogenic mutation in *SMAD3*. Testing of the proband's family members lead to identification of a serious health risk for thus far two additional family members. Identification of the *SMAD3* mutation in the proband's father also explains his orthopedic complications. While other congenital heart defects such as persistent ductus arteriosus, atrial septal defect, and pulmonary valve stenosis have been observed in patients with *SMAD3* mutations, to our knowledge, this is the first case reported with HLHS.

The findings in this family are consistent with the phenotype previously described in patients with AOS. The majority of previously described mutations in *SMAD3* were observed in families with TAAD, implying a bias towards an aortic aneurysm and dissection phenotype [10, 11]. The patients in this family were ascertained only after screening in the proband, who developed significant neoaortic aneurysm following extensive cardiac surgical history for his congenital heart defect.

The phenotype of AOS in this family is age dependent with the 46-year-old father exhibiting both vascular and skeletal features while the 9-year-old brother has neither. This is consistent with many previous reports; however, a recent study by Wischmeijer et al. reported pathologic aortic dilation in a patient as young as 12 months [12]. The report by Hilhorst-Hofstee et al. of an unanticipated copy number variant of chromosome 15 disrupting *SMAD3* revealed a three generation family at risk for aortic dissection. This included the 12-year-old proband initially investigated for mild mental retardation and a 4-year-old asymptomatic cousin. The two children in this family had the abnormal feature of ascending aorta wider than the sinotubular junction; the authors queried if this may be the first sign of future dilation [13]. In our case, the proband's 9-year-old brother (whose transthoracic echocardiogram and MRA were normal) also had an ascending aorta wider than the sinotubular junction (ascending aorta = 2.4 cm, sinotubular junction = 2.2 cm).

Aortic dilations in congenital aortic diseases such as bicuspid aortic valve and coarctation of the aorta are well described, and of the cyanotic congenital heart diseases, Tetralogy of Fallot has been the most widely studied with dilation at the annulus and sinus occurring in 88% and 87% of patients respectively [14]. Neoaortic root dilation has also been observed in other CHD's including transposition of the great arteries following aortic switch operation, [15, 16] following Ross procedure for congenital aortic valve disease, [17] and truncus arteriosus [18]. Specific to our case, neoaortic root dilation and valve regurgitation were reported in a group of patients with HLHS following staged palliation by Cohen et al. [19] in their cohort of 53 patients; aortic root dilation and aortic valve regurgitation were observed in 98% and 61%, respectively. Transection of the main pulmonary artery followed by reconstruction, exposing the neoaortic root (native pulmonary root) at systemic pressure, is the common denominator for all surgical interventions where the pulmonary valve becomes the neoaortic valve. It has been suggested that under these circumstances, aortic dilatation could be a result of blood flow to the vessel wall being compromised [19].

While neoaortic root dilation and aortic valve regurgitation are commonly observed in HLHS patients following staged palliation, the degree of aneurysm observed in our patient is not commonly observed in this patient cohort. Additionally, significant aneurysm formation leading to dissection is not typical based on the literature. Aortic dissection was reported in one 26-year-old male with HLHS where the aortic root measured 7.8 cm by transthoracic echocardiogram [20]. Additional information regarding the phenotype of this patient or his family history was not reported.

The phenotype of the proband and his father is consistent with what has been described thus far for the aneurysms-osteoarthritis syndrome, which leads us to conclude that the c.3G>A mutation in *SMAD3* found in this family is pathogenic and is an explanation for their clinical findings. Identification of AOS in this family allowed for the screening of at risk family members, identification of early vascular disease, and it offers an explanation for the aneurysmal history in the proband and degenerative disk disease and arthritic history in his father. This case highlights the importance of screening patients with congenital heart defects for genetic causes of known aneurysmal disease.
